# Treatment of fistula in-ano with fistula plug: experience of a tertiary care centre in South Asia and comparison of results with the West

**DOI:** 10.1186/s13104-018-3641-x

**Published:** 2018-07-28

**Authors:** Isuru S. Almeida, Dakshitha Wickramasinghe, Pragathi Weerakkody, Dharmabandhu N. Samarasekera

**Affiliations:** Department of Surgery, Faculty of Medicine, University of Colombo, P.O. Box 271, Kynsey Road, Colombo 8, Sri Lanka

**Keywords:** Fistula-in-ano, Fistula plug, South Asia

## Abstract

**Objectives:**

Surgery for fistula in ano is associated with anal incontinence. The biologic anal fistula plug (AFP) can minimize this. This is a retrospective analysis of patients with cryptoglandular anorectal fistulae, who underwent a surgical procedure using AFP. Patient’s demographics and characteristics of the fistulae were obtained from a prospective database. Each primary opening was occluded by using an AFP. Success was defined by the closure of the external opening and absent drainage.

**Results:**

Fifty-one patients were treated with AFP (male:female: 37:14), mean age 42 years (SD ± 14.86, range 26–70). Ten patients defaulted follow-up. Forty-seven procedures were analysed. Twenty-three (56.1%) patients had complete healing while 18 (43.9%) patients failed the fistula plug procedure during the follow up period of 12 months. Logistical regression failed to identify any statistical significant association with demographic or disease factors and healing. Healing was 1.5 times less likely for every failed procedure prior to AFP insertion. Contrary to other published studies, placement of fistula plug was associated with much lower overall rates of fistula healing. Highest success rates were seen in simple fistulae when compared to the complex type. Repeat plug placement may be successful in selected patients.

## Introduction

The management of anal fistulae is a challenge for the colorectal surgeon. The goal of fistula surgery is to cure the fistula while preserving anal continence. The prevalence of fistula in ano is estimated to be 1 in 10,000 individuals [1]. Men are twice as likely to develop fistulae than women and they typically present at a mean age of 40 years [2]. Fistulotomy has become a less widespread procedure because of the risk of incontinence up to 17% [3]. Minor disturbance to anal continence following surgery for fistula-in-ano occurs in 18–52% of the patients [4, 5] and major incontinence affects 6.7–44% [5–7]. However sphincter sparing procedures like treatment with fibrin glue are often unsatisfactory and may contribute to a decreased quality of life and persistence of the fistula [8].

Treatment modalities like the cutting seton is effective in up to 70% of the patients but has a risk of minor incontinence in 36% and major incontinence in approximately 12% [9]. In 2006, the anal fistula plug (AFP) was introduced as a “sphincter saving” procedure [10] and an alternative to open surgery. Surgisis^®^ AFP is a bio absorbable xenograft made of lyophilized porcine intestinal sub-mucosa [11]. The material has an inherent resistance to infection, produces no foreign body or giant cell reaction and becomes repopulated with host cell tissue during a period of three months [12, 13]. Surgisis^®^ AFP™ was developed with the aim of providing a quick, safe and convenient treatment for anal fistulae [14]. The initial series reported a success rate of 87% when compared with a 40% success rate with fibrin glue at a mean follow up of 2-years [15]. Subsequent studies have reported widely variable heal rates for AFP, ranging from 43% [16] to 86% [17]. Available knowledge also indicates that the placement of Surgisis^®^ AFP™ is more successful in fistula with a single primary opening that in complex fistulae [8]. Since there have been very few studies conducted in South Asia on the use of AFP, we present the results of a single institution using AFP for treatment of fistula in ano.

The aim of this retrospective study was to assess the efficacy of the AFP in the treatment of cryptoglandular anorectal fistula.

## Main text

The study was conducted as a retrospective review of consecutive patients who underwent AFP insertion at the University Surgical Unit, the National Hospital of Sri Lanka between January 2007 to January 2016. Data were retrieved from a prospectively collected database. Those who defaulted follow up and followed up by other centres were excluded from the study. Fifty-one patients who had a fistula secondary to cryptoglandular abscess were enrolled. Patients with inflammatory bowel disease were not considered for AFP insertion. Patient selection, AFP insertion and postoperative evaluation were performed by a single colorectal surgeon. All patients gave informed consent for the placement of AFP. In patients with multiple extensions, the AFP was placed at the primary tract. Demographic data, fistula characteristics and post-operative outcome were recorded.

All patients had a drainage seton inserted for a minimum of 6 weeks prior to AFP insertion to minimize the sepsis. Procedures were performed under spinal anaesthesia with the patient placed in lithotomy position. All patients received intravenous metronidazole 500 mg and co-amoxycalv 1.2 g before the procedure. All fistula tracts were identified and the primary opening was located by using conventional fistula probe and/or by injection of hydrogen peroxide. If a seton was present in the tract previously, this was removed. All the tracts were curetted of granulation tissue was sent for histology. After soaking in a 0.9% NaCl solution, the AFP was inserted from the internal opening and fixed at the internal opening with 2-0 polyglactin stitches, thereby closing the internal opening. Any excess plug material, which was not implanted in the tract, was removed and the plug was anchored to skin. The external openings were left open, enabling further drainage from the fistula tract and to minimise infection or abscess formation. Gentamicin and providone-iodine solutions were avoided as recommended by the manufacturer.

Patients were followed up every 3 weeks until the wounds healed, and at three and six month intervals until 12 months. Each follow up visit included an interview and a physical examination. Success was defined by the closure of the all external openings and absent perianal drainage at the last follow up (3, 6 or 12 months). Recurrences was considered as a treatment failure.

Statistical analysis was done using SPSS^®^ 21.0 statistical software (SPSS Inc., USA). The results of categorical variables were expressed as frequencies and proportions while continuous variables were expressed using means ± standard deviations. The Chi square test was used to compare data between groups. A *p* value < 0.05 was considered to be statistically significant.

Fifty-one patients underwent anal fistula plug insertion. However, 10 patients defaulted follow up and were excluded from the study. The 41 patients included in the final analysis had 27 (65.9%) males. The mean age was 42 (range 26–70, SD ± 14.87) years. Simple fistulae were detected in 32 (78.0%) patients. Trans-sphincteric fistulae were the commonest type (n = 30, 73.2%).

Seven patients underwent multiple AFP insertion procedures. Therefore, there were a total of 48 procedures. Four out of the 7 who had a 2nd AFP inserted were cured with the second fistula plug insertion.

Cumulatively, the patients had had 45 procedures done prior to the placement of AFP (Table 1).

**Table 1:** Fistula characteristics

		n
Fistula type	Simple	32 (78%)
	Complex	9 (22%)
Internal opening level	Below dentate line	14 (34.1%)
	At the dentate line	24 (58.5%)
	Above the dentate line	3 (7.3%)
External opening number	1	31 (75.6%)
	2	7 (17.1%)
	3	3 (6.3%)
External opening position	Anterior	23 (56.1%0
	Posterior	18 (43.9%)
Procedure	Seton (dranage/cutting)	27
	Fistulotomy	9
	Fistulotomy and Seton	9

Overall fistula healing rate was seen in 23 (56.1%) patients. The median follow up was 6.7 (range 1–23) months post operatively. Dislodgement of the fistula plug was thought to be the cause for failure in most cases. Six recurrences (14.6%) were identified at a follow up of 3 months.

Tables 2 and 3 describe the gender distribution and the anatomy of the primary tract, respectively.

**Table 2:** Gender distribution of clinical outcome of Surgisis^®^ AFP™ and fistula type

Clinical outcome of Surgisis^®^ AFP™	Type of fistula	Male (n = 27)	Female (n = 14)
Succeeded	Simple	17	7
Succeeded	Complex	3	2
Failed	Simple	4	4
Failed	Complex	3	1

**Table 3:** Primary tract of fistulae with the outcome at follow up

Primary tract	Healed (n = 29)	Non-healed (n = 12)	*p* value*
Trans-sphincteric(n = 30)	21	9	0.242
Inter-sphincteric(n = 10)	8	2	
Superficial (n = 1)	–	1	

*x^2^-test

During the follow up period, 23 (56.1%) plug placements out of 47 total procedures were successful. This included 15 (36.6%) patients in whom the fistula healed with the insertion of an AFP and 8 (19.5%) after the insertion of the 2nd AFP.

Patients who had the fistula healed with the insertion of an AFP had a lower number of median procedures prior to insertion of an AFP (2 vs 5, Mann–Whitney U 58.5, p = 0.001).

A logistic regression was performed to identify the factors independently associated with successful healing of the fistula following AFP insertion. Even though the model was statistically significant (p = 0.002), it only explained 29.3% of the variance observed and predicted 72.5% of the cases accurately. The only factor showing a statistically significant association with non-healing was the mean number of procedures prior to having a fistula plug inserted, with healing being 1.5 times less likely with each previous procedure.

The sex of the patient, use of a seton prior to AFP insertion, type of the fistula (simple vs complex), location or level of the internal opening, type of fistula tract, site of external opening, presence of abscesses or having an AFP inserted as the first procedure showed no statistically significant association with healing.

There are many surgical options available for the treatment of fistula in ano and all are based on 3 main principles; (a) control of sepsis (b) closure of the fistula and (c) maintenance of continence [18]. At least, as far as the quality of life is concerned, the latter is as important as the former. The AFP is able to achieve all 3 of these due to its inherent qualities. The AFP is resistant to infection [12] and therefore aids in controlling the sepsis. It provides a scaffolding for the host tissue to grow and facilitates healing of the fistula. It also eliminates the need for surgery that can damage the anal sphincter and thereby minimizes the risk of incontinence. Sphincter sparing procedures like fibrin glue was popularised to reduce the incident of postoperative incontinence after these procedures [19–21]. However, even though the initial results were promising for the fibrin glue with long term run, the results were disappointing [22]. The fistula plug was developed to be bio absorbable and resistant to infection [23]. This, therefore, provided an attractive alternative for patients with anal fistulae.

When comparing with previous research [14, 15], our findings show a marked disparity between the West and Asia related to fistula healing rates with the AFP. In a similar retrospective analysis of 45 patients, Alex et al. [5] showed a 84% healing rate in 3–8 weeks postoperatively with the AFP. However, other studies have shown much lower success rates [24–27]. Fifty six percent of our patients had complete healing after the first procedure, which is lower than previously described healing rates [15]. However, there are studies that have reported much lower healing rates [24]. The relatively high success rate of our study is perhaps contributed by the fact that it was a single surgeon who performed all the surgeries. Previous studies which had fewer surgeons (1 surgeon—70.8% [8] and 66% [5], 2 surgeons—86% [28]) have reported higher success rates than studies with a higher number of surgeons (5 surgeons—43% [16], 6 surgeons—44% [25]). This may be because of the difference in experience and the competence of different surgeons. Findings of Han et al. [29] (though the study used an acellular dermal matrix plug) has also identified treatment by a non-expert surgeon is associated with plug failure.

Sentovich et al. [30] suggested that the degree of scarring and fibrosis from previous procedures might contribute to poor healing of fistulae following fibrin glue treatment. It is difficult to comment whether a similar observation can be made following treatment with the fistula plug. Scarring and fibrosis from previous surgery might contribute to the early dislodgement of fistula plug [25]. Our findings also suggest that patients who have had fewer previous procedures are more likely to benefit from AFP placement.

Another promising treatment option for recurrent fistula-in-ano is the injection of autologous stem cells to the fistula tract, followed by the closure of the internal opening [31]. Garcia-Olmo et al. reported a 60% long-term cure rate, with minimal impact on continence.

Since males are more affected than females (65.9 vs 34.1%) and relatively young population has been affected (median age—42 years), fistula in ano negatively affects the economy of the country as well. Although the AFP imposes a higher initial cost, there is an overall cost saving [5]. Therefore, a proper patient assessment should be done prior to the placement of an AFP to reduce failure rates.

The AFP was introduced as an alternative treatment for simple perianal fistulae of cryptoglandular origin with no impact on sphincter function. However, we only saw healing in just over half of our patients. However, since some patients had the fistula healed after repeated AFP insertion, the failure of AFP once should not be a contraindication for repeat AFP insertion. Early AFP insertion appears to be more successful than when utilized after multiple failed procedures.

## Limitations

The results of this study should be interpreted with some caution as, 20% of patients defaulted the follow up. No formal assessment of anal sphincter function was done in our patients and we consider it to be another drawback of our study. Another possible drawback is that we did not confirm fistula healing using endoanal ultrasound or Magnetic Resonance Imaging (MRI). Given the logistical and financial limitations of a developing country and that the aim of treatment is cessation of discharge and healing the fistula rather than radiological healing, we feel that this does not affect our findings.
